# Par-complex aPKC and Par3 cross-talk with innate immunity NF-κB pathway in epithelial cells

**DOI:** 10.1242/bio.20135918

**Published:** 2013-10-08

**Authors:** Radia Forteza, Flavia A. Wald, Anastasia Mashukova, Zhanna Kozhekbaeva, Pedro J. Salas

**Affiliations:** Department of Cell Biology, University of Miami Miller School of Medicine, 1600 NW 10th Avenue, Miami, FL 33136, USA

**Keywords:** Epithelial polarity, Par-complex, Atypical PKC

## Abstract

Components of the Par-complex, atypical PKC and Par3, have been found to be downregulated upon activation of NF-κB in intestinal epithelial cells. To determine their possible role in pro-inflammatory responses we transduced Caco-2 human colon carcinoma cells with constitutively active (ca) PKCι or anti-Par3 shRNA-expressing lentiviral particles. Contrary to previous reports in other cell types, ca-PKCι did not activate, but rather decreased, baseline NF-κB activity in a luminiscence reporter assay. An identical observation applied to a PB1 domain deletion PKCι, which fails to localize to the tight-junction. Conversely, as expected, the same ca-PKCι activated NF-κB in non-polarized HEK293 cells. Likewise, knockdown of Par3 increased NF-κB activity and, surprisingly, greatly enhanced its response to TNFα, as shown by transcription of IL-8, GRO-1, GRO-2 and GRO-3. We conclude that aPKC and Par3 are inhibitors of the canonical NF-κB activation pathway, although perhaps acting through independent pathways, and may be involved in pro-inflammatory responses.

## Introduction

Epithelial apico-basal polarity is controlled by signaling complexes such as the apical aPKC-Par6-Par3 (Par-complex, where atypical PKC comprises PKCζ and PKCι/λ isoforms). Recently, we found a steep down-regulation of aPKC and Par3 (Bazooka in Drosophila, not to be confused with Protease Activated Receptors) downstream of a common effector of innate immunity and pro-inflammatory signaling, NF-κB, in human intestinal epithelial cells. It was demonstrated in Caco-2 cells (human colon carcinoma) in culture and in an animal model of colitis (Mashukova et al., 2011), as well as a negative correlation of aPKC expression with inflammation in enterocytes from Inflammatory Bowel Disease (IBD) patients (Wald et al., 2011). This effect on the Par-complex downstream of a pathway, previously thought to be independent, is important because chronic NF-κB activation is part of the mechanism that contributes to barrier (i.e. tight junction) opening in IBD (Rogler et al., 1998; Wullaert et al., 2011; Xavier and Podolsky, 2007). The functional implication is that the Par-complex is key to the organization of tight junctions (Wang and Margolis, 2007). The aPKC-Par6-Par3 complex is localized to tight junctions and the apical domain in polarized epithelia. This exquisite localization contrasts with a broad cytoplasmic and nuclear localization in non-polarized cells (Stross et al., 2009). The Par-complex is linked by Par6 (Joberty et al., 2000), which binds aPKC through an N-terminal PB1 domain and Par3 through a PDZ domain. The PB1-mediated Par6 binding is essential for localization and activation of aPKC (Graybill et al., 2012). An interaction between aPKC and Par3 through the kinase domain is dynamic, as Par3 direct phosphorylation in Ser827 by aPKC results in Par3 *activation* and dissociation from Par6-aPKC (McCaffrey and Macara, 2009). Par3 is a scaffolding protein with multiple interactions (Tepass, 2012). However, to our knowledge, no connection with innate immunity pathways has been described for Par3 so far.

The PKCζ and PKCι/λ knock-out mice highlighted a role of aPKC activating NF-κB (Frey et al., 2006; Martin et al., 2002; Sajan et al., 2009a; Sajan et al., 2009b; Win and Acevedo-Duncan, 2008; reviewed by Diaz-Meco and Moscat, 2012). The mechanisms for NF-κB activation by aPKC involve IKKαβ phosphorylation with the ensuing IκB degradation (Win and Acevedo-Duncan, 2008) as well as direct phosphorylation and activation of relA(p65) by aPKC (Duran et al., 2003). Bearing in mind the effects of pro-inflammatory stimuli on the Par-complex proteins aPKC and Par3 in intestinal cells, we asked whether these proteins may, in turn, participate in the control of epithelial pro-inflammatory responses. The results showed an antagonistic effect of Par3-aPKC with pro-inflammatory signaling in epithelial cells. Such a role is contrary to the predicted function for aPKC.

## Results

### Expression of constitutively active PKCι fails to induce NF-κB activation in intestinal epithelial cells

**Fig. 1.**
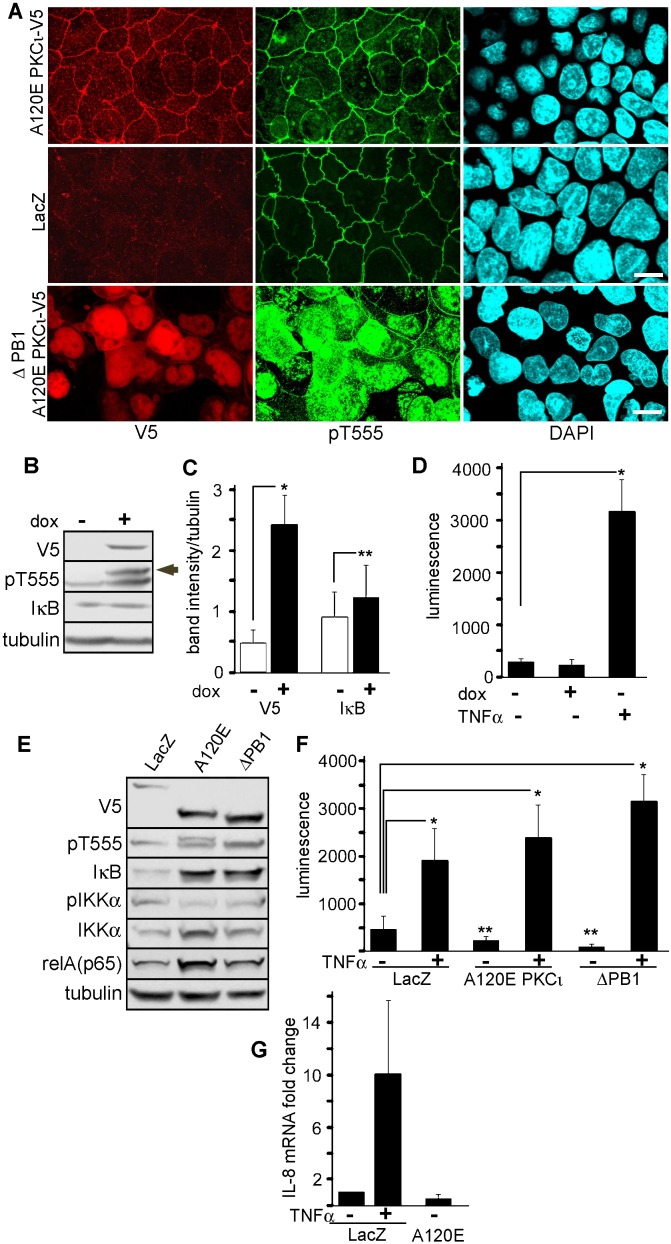
Expression of constitutively active PKCι fails to activate NF-κB in intestinal epithelial cells. (A) Caco-2 cells transduced with lentiviral particles expressing V5-tagged ca-A120E-PKCι, ca-ΔPB1A120E-PKCι, or LacZ (control) and selected with blasticidin were fixed at 7 days after confluency. Bars, 10 µm. (B–D) Caco-2 cells were transduced with similar lentiviral particles expressing A120E-PKCι under a tetracycline-inducible promoter. Expression was induced with 2 ng/ml doxycycline (dox). (B) Immunoblot from parallel cultures confluent for 10 days and induced (+) or not (−) for 24 hours. The V5-tagged PKCι product can be observed as a slightly higher molecular weight band (arrow). (C) Quantification of signal from independent experiments similar to B. Matched samples t test: * *P*<0.001, ** *P*<0.05, *n* = 7. (D) Experiments similar to those shown in B–C were conducted but the cells were further transduced with lentiviral particles expressing luciferase under a NF-κB-inducible promoter and puromycin resistance. After puromycin selection, cells confluent for 10 days on filters were induced with doxycyclin or not, or stimulated with basolateral TNFα for 24 hours. * *P*<0.05, *n* = 3. (E) Caco-2 cells were transduced and selected to express LacZ (control), A120E-PKCι (A120E), or ΔPB1-A120E-PKCι (ΔPB1) and analyzed by immunoblot. (F) Similar cultures were stimulated or not with TNFα and NF-κB activation was measured by a luciferase transcriptional reporter. * *P*<0.05, ** *P*<0.025, *n* = 3. (G) Expression levels of IL-8 mRNA were measured by RT-qPCR in LacZ- and A120E-PKCι-expressing cells and expressed as fold change respect to the non-treated (−) control LacZ sample.

To test the hypothesis that Par-complex aPKC regulates NF-κB activity in epithelia, we prepared two C-terminal V5-tagged constitutively active (ca) constructs as follows. A120E-PKCι is a ca-mutant with a non-functional internal inhibitory pseudosubstrate (Spitaler et al., 2000). Second, we also deleted the entire PB1 domain (aa 29–108), in the same ca-A120E background (hereafter refered to as ΔPB1 mutant). This mutant was meant to differentiate the scaffolding functions of the PB1 interaction (i.e. Par6 binding) from its kinase regulatory function. In other words, the ΔPB1-A120E-PKCι is active but unable to bind Par6. A120E-PKCι stably transduced with lentivirus in Caco-2 cells, localized normally to tight junctions and the apical domain, just like the endogenous aPKC. The ΔPB1-A120E-PKCι mutant, conversely, showed a diffuse cytoplasmic and nuclear distribution (Fig. 1A). This nuclear redistribution was to be expected since PKCι contains nuclear localization signals (Perander et al., 2001). It confirmed that aPKC localization to the tight junctions and the apical domain is strongly dependent on its PB1 domain interactions.

**Fig. 2.**
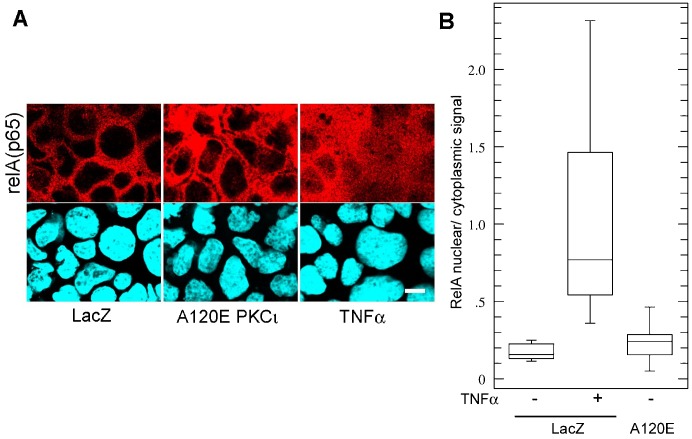
Expression of constitutively active PKCι fails to induce RelA nuclear translocation. (A) Caco-2 cells constitutively expressing LacZ (control) or ca-A120E-PKCι were analyzed for relA(p65) immunofluorescence. LacZ-expressing cells were used as a positive control. Bar, 10 µm. (B) Quantification of average intensities of nuclear/cytoplasmic pixels from experiments as described in A, graphed as “whisker” boxes (LacZ, 19 cells; LacZ TNFα, 19 cells; A120E-PKCι, 31 cells). There was no statistical difference between LacZ and A120E cells.

First, we transduced A120E-PKCι under a tetracyclin-inducible promoter. Doxycycline induced the expression of A120E-PKCι at levels similar to those of the endogenous aPKC as determined by comparing phosphorylation of the turn domain (pT555) (Fig. 1B, higher band, arrow shows the V5-tagged mutant; we used anti-pT555 antibodies because they recognize the active conformation of both PKCι and PKCζ, i.e. total active aPKC). However, A120E-PKCι failed to activate NF-κB as determined by IκB levels and a luciferase reporter assay (Fig. 1B–D): IκB significantly increased rather than decreasing (Fig. 1C), and A120E caused no increase in luciferase expression (Fig. 1D). TNFα stimulation was used as a positive control (Fig. 1D). Then, similar experiments were also conducted in Caco-2 cells stably transduced with either LacZ (control), A120E-PKCι, or ΔPB1-A120E-PKCι. Expression of both ca-mutants resulted in increased steady-state levels of IκB and relA(p65), and no changes in IKKα phosphorylation (Fig. 1E). Furthermore, both ca mutants failed to stimulate NF-κB as indicated by the luminescence reporter (Fig. 1F). Instead, there were significant decreases in of luciferase expression (65 and 90%) in the ca-PKCι-expressing cells as compared to LacZ-expressing cells. This suggests a steep decrease in the baseline activity of NF-κB. Conversely, ca-PKCι expression failed to affect the response to TNFα as these responses were not statistically different form each other (Fig. 1F). These results were further confirmed by IL-8 mRNA transcription, which did not change in A120E-PKCι-expressing cells (Fig. 1G). In addition, we also determined RelA(p65) nuclear translocation in A120E-PKCι-expressing Caco-2 cells. In TNFα-stimulated cells RelA signal appeared in the nucleus and cytoplasm (signal ratios near 1), while in non-stimulated cells or in A120E-PKCι-expressing cells the nuclei remained mostly negative for relA, with nuclear/cytoplasmic ratios around 0.2. These low ratios were not statistically different from each other (Fig. 2). In summary, ca-aPKC failed to cause RelA nuclear translocation.

### Constitutively active A120E PKCι activates NF-κB in mesenchymal HEK cells

**Fig. 3.**
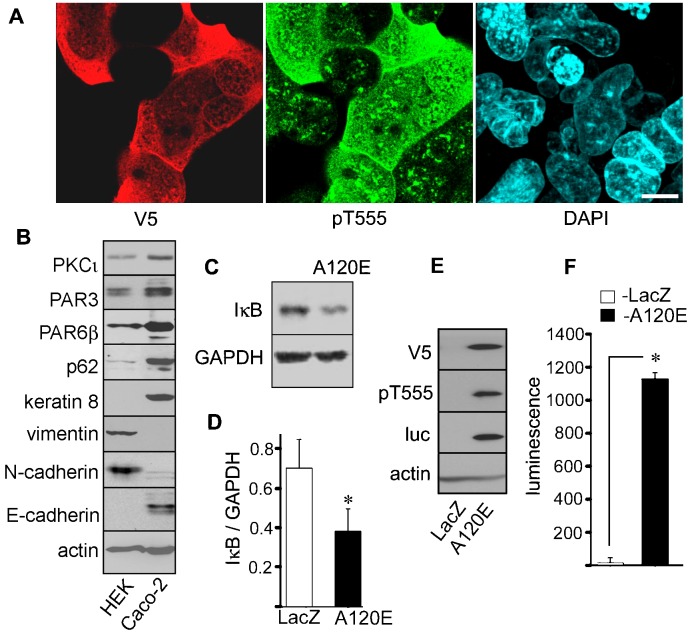
Expression of constitutively active A120E-PKCι in HEK293 cells activates NF-κB. (A) HEK293 cells were transduced with the same lentiviral particles expressing A120E-PKCι or LacZ as in Fig. 1, and selected. The cells were cultured for 3 days, fixed, and processed for V5-tag (red channel) or pT555 aPKC (green channel). Bar, 10 µm. (B) Confluent cultures of non-transduced Caco-2 and HEK293 cells were analyzed by immunoblot with the antibodies indicated on the left side. (C) HEK293 cells, constitutively expressing LacZ or A120E-PKCι were analyzed by immunoblot (D) Quantification of IκB was performed in 3 experiments as described in C. * *P*<0.025. (E) Similar cultures expressing LacZ or A120E-PKCι were transduced with lentiviral particles expressing a luciferase (luc) NF-κB transcriptional reporter and puromycin selected. The cells were analyzed by immunoblot with the antibodies indicated on the left side. (F) Quantification of luciferase luminescence in 3 experiments as described in E. * *P*<0.001.

To rule out the possibility of unnoticed mutations in the ca-PKCι constructs as a trivial explanation for the negative results in Caco-2 cells, we decided to confirm the biological activity of our ca-PKCι mutants. Thus, we transduced human embryonic kidney (HEK293) cells with the same lentiviral vectors used in Figs 1 and 2. In these cells aPKC is known to activate NF-κB (Sanz et al., 1999). Unlike in Caco-2 cells, HEK cells expressing A120E-PKCι showed a diffuse cytoplasmic and nuclear localization with minimal localization to the cell-cell contact (Fig. 3A). HEK293 cells expressed other components of the Par-complex, such as Par3 and Par6 (Fig. 3B), but displayed a mesenchymal phenotype, with expression of vimentin and N-cadherin, instead of keratins or E-cadherin. Both cell types expressed the cognate aPKC PB1 binding proteins, Par6 and p62 (Fig. 3B). Therefore, the lack of localization of A120E-PKCι may be due to the absence of tight junctions in HEK293 cells. Stable expression of the ca-PKCι resulted in a sustained and significant decrease in IκB (Fig. 3C,D). Importantly, expression of A120E-PKCι strongly induced the expression of reporter luciferase (Fig. 3E,F, luc). Altogether, these data indicate that PKCι inhibits the NF-κB pathway in Caco-2 epithelial cells, an opposite effect to its action in other cells, including HEK293. This inhibition is independent of PB1 scaffolding interactions and PKCι subcellular localization.

### Par3 inhibits NF-κB activation downstream of the TNFR pathway

**Fig. 4.**
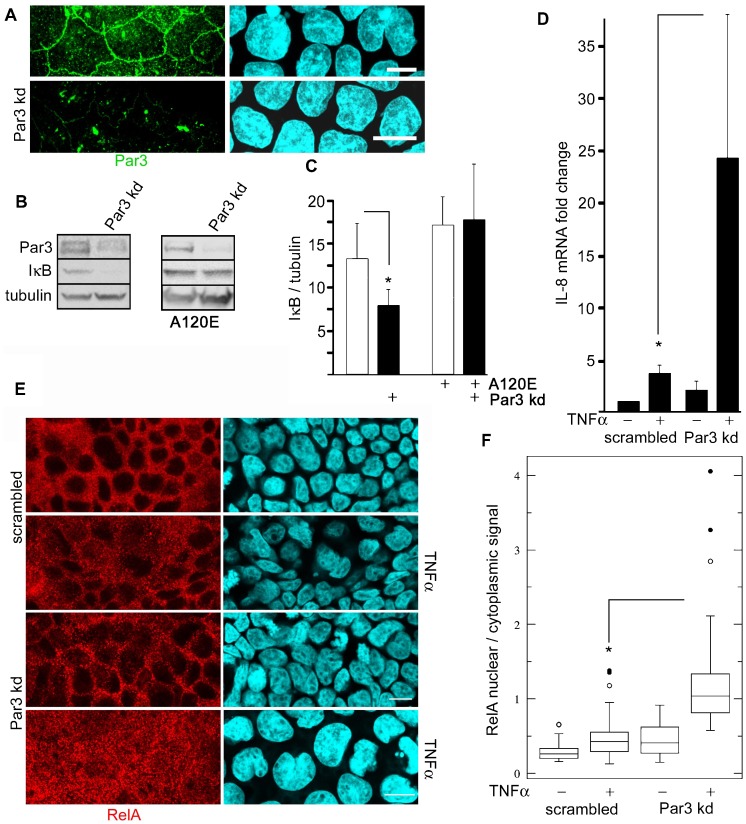
Par3 modulates TNFα response. (A) Caco-2 cells were transduced with lentivirus expressing anti-Par3 shRNA (kd) and visualized by immunofluorescence. Bars, 10 µm. (B) Similar cultures were analyzed by immunoblot, in cells with or without stable ca-PKCι expression (A120E). (C) Quantification of the IκB bands in cells expressing or not Par3 shRNA, relative to tubulin signal (*n* = 4; * *P*<0.025), and A120E-PKCι (*n* = 5). All the values were normalized for tubulin signal. (D) IL-8 mRNA was measured by RT-qPCR in scrambled shRNA-transduced and Par3 kd cultures previously incubated for 24 hours in TNFα (+) or control (−). Results are expressed as fold changes respect to the mock control levels. * *P*<0.025 (*n* =  3). (E) Immunofluorescence determination of subcellular distribution of relA(p65) in Caco-2 cells grown on filters and stimulated or not with basolateral TNFα. Bars, 10 µm. (F) Quantification of average intensities of nuclear/cytoplasmic pixels from experiments as described in E, graphed as “whisker” boxes (scrambled, 39 cells; scrambled TNFα, 41 cells; Par3 kd, 33 cells, Par3 kd TNFα, 27 cells; * paired comparison *P*<0.001). Suspected outlier points (beyond the third quartile) are shown individually.

**Fig. 5.**
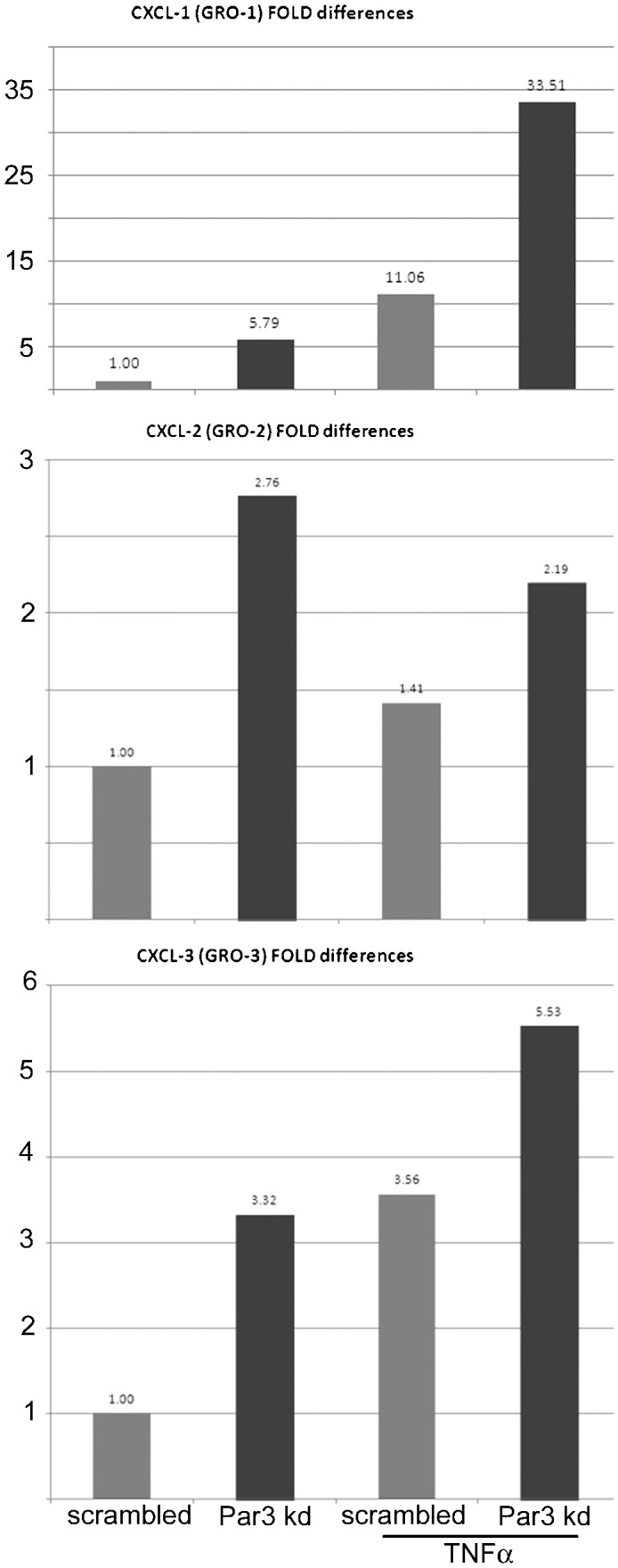
Par3 kd increases other mRNAs transcriptionally regulated by NF-κB. Caco-2 cells transduced and treated with TNFα as described in Fig. 4, were used to extract RNA. mRNAs from NF-κB-regulated GRO-1, GRO-2, and GRO-3 were measured by RT-qPCR. Grey bars represent values from cells expressing a scrambled shRNA, black bars, mRNAs from cells expressing specific anti-Par3 shRNA. The fold differences were normalized to the values of scrambled-expressing control cells for each transcript.

Because Par3 is activated by aPKC (St Johnston and Ahringer, 2010), and it is also down-regulated upon pro-inflammatory stimulation (Mashukova et al., 2011), we expected that Par3 knockdown (kd) would have an opposite effect and rescue ca-PKCι effect on NF-κB. The lentiviral-delivered shRNA kd efficiency was approximately 60% (Fig. 4A,B). As expected, Par3 kd resulted in a sustained significant reduction of IκB (Fig. 4B,C). However, Par3 kd failed to rescue the effect of da-PKCι expression on IκB (Fig. 4C). In fact, Par3 kd lead to a 2.1 fold increase in IL-8 mRNA transcription as compared with scrambled shRNA-expressing cells, indicating NF-κB activation. This increase in IL-8 was approximately half the maximal IL-8 transcription induced by TNFα (Fig. 4D). However, the response to TNFα in Par3 kd cells was 5 fold higher than in equally stimulated cells expressing scrambled shRNA. To independently confirm this result, similar experiments were conducted determining relA(p65) nuclear translocation. In unstimulated cells nuclei appeared as a negative image for relA signal (Fig. 4E). In scrambled shRNA cells, TNFα stimulation increased the nuclear/cytoplasmic signal ratio by 2 fold. In Par3 kd cells, basolateral TNFα increased the median ratio 4 fold, with cells in the third quartile reaching nuclear/cytoplasmic values around 2 (Fig. 4F). To analyze the downstream transcriptional activation, we further analyzed 3 additional mRNAs known to be under NF-κB control: GRO-1, GRO-2 and GRO-3. The results were similar to those of IL-8 transcription, although there was quantitative variability. GRO-1 showed fold ratio increases in the same range as IL-8, while GRO-2 and -3 showed more modest fold ratio increases, in the range 3–6. Importantly, for all three GRO mRNAs, Par3 kd cells showed increased transcription, and, for GRO-1 and -3, the TNFα response was also stronger in Par3 kd than in scrambled-expressing controls (Fig. 5). These results are fully consistent with the IL-8 data shown in Fig. 4.

We conclude that Par3 kd greatly enhances NF-κB activation by TNFα. While Par3 is inhibitory of NF-κB, the lack of rescue of ca-PKCι expression and the differential effect on the TNFR pathway suggest a mechanism that is independent of, but synergistic with aPKC.

## Discussion

Our results indicate that both aPKC and Par3 are antagonistic of pro-inflammatory signaling. The inhibitory effect of PKCι on NF-κB activation described here is likely not present in malignant cells where aPKC expression acts as an oncogene, because in those cases NF-κB transactivation is necessary for the malignant phenotype (Lu et al., 2001).

Because HEK293 and Caco-2 cells express the same Par-complex proteins (Par3, Par6 and aPKC) and PB1 domain binding partners (p62 and Par6) (Fig. 3B), the difference between both cell lines must be downstream of the Par-complex. This conclusion is highlighted by the fact that the ca-PKCι with a deleted PB1 domain, which clearly did not incorporate into the Par-complex in Caco-2 cells, had a similar effect on NF-κB as the normally localized kinase. Both ca-PKCι expression or Par3 knock-down induce sustained changes in IκB steady-state levels and relA(p65) nuclear translocation, suggesting that the effect of Par-complex proteins takes place via the canonical activation mechanism for NF-κB. Hence, the data is consistent with the interpretation that a “switch” mechanism determining NF-κB inhibition in the epithelium (as opposed to activation in other cells) is upstream of relA(p65) cytosolic retention. In addition, we also conclude that Par3 effect on the TNFR pathway is likely independent of aPKC activation. It is of note that Par3 is known to interact with Gab1, which is also involved in epithelial polarity (Yang et al., 2012). In turn, Gab1 participates in the activation of NF-κB by TNFα in endothelial cells (Che et al., 2002).

The molecular switch or switches that control the activating or inhibitory effect of aPKC on NF-κB in different cell types and the molecular relationship between Par3 and the TNFR pathway will require additional investigations and may have profound implications not only for epithelial response to pro-inflammatory stimuli, but also for diseases involving the aPKC – NF-κB axis such as diabetes and obesity (Sajan et al., 2009a; Sajan et al., 2009b). The functional implication of these mechanisms is that the the Par-complex may modulate responses that include secretion of cytokines in addition to the well-known tight junction opening (Pasparakis, 2012), an as yet unsuspected possible mechanism in chronic inflammation.

## Materials and Methods

### Lentivirus constructs and expression vectors

Lentiviral constructs expressing shRNA and puromycin resistance were obtained from Sigma (anti-Par3, TRCN 0000118134, scrambled non-mammalian shRNA SHC002V). Human PKCι ORF was obtained from Origene (SC118455 NM_002740) The mutagenesis of A120E-PKCι was achieved by PCR using modified primers (Kadowaki et al., 1989). Deletion of the PB1 domain was performed by primer extension PCR (Ho et al., 1989) using the A120E mutant as template. The mutants were verified by PCR sequencing. For protein expression, these constructs were cloned in a pLenti6.3/V5-DEST™ Gateway® Vector (K5330-Life Technologies), and packaged using ViraPower™ HiPerform™ Lentiviral Expression Systems (blasticidin resistance) (K5310-00, Life Technologies). For tet-inducible expression of PKCι-A120E, the cells, transduced and selected as described above, were passed 1:4, immediately transduced with lentiviral particles expressing TR, and selected in geneticin in addition to blasticidin. This order of transduction was found to be essential. Tet-responsiveness in double transduced cells lasted just for 2–3 passages and was determined in each passage in parallel cultures before experiments.

### Antibodies and reagents

Antibodies used in this study were as follows (supplier, cat. #): IκB (Cell Signaling, 9246S), IKKα and p-IKKα (Invitrogen), PKCι (BD Biosciences, 610175), relA(p65) (Cell Signaling, 3987), pT555 (Abcam, ab5813; Genetex, GTX25813), V5 epitope (Invitrogen, r96025), PAR3 (Millipore, 07-330, PAR6β (Abcam, ab49776), p62 (Cell Signaling, 51145), keratin 8 (TROMA 1, Developmental Studies Hybridoma Bank), vimentin (Epitomics, 4211-1), N-cadherin (Abcam, ab18203), E-cadherin (BD Transduction, 610181), actin (MP Biomed, 691001), tubulin (Sigma, T6199), GAPDH (Sigma, G9545), luciferase (GeneTex, GTX72301). TNFα (R&D) was always added from the basolateral side in Transwell inserts at a final concentration of 20 ng/ml and incubated overnight.

### Luminescence

Triplicates of Caco-2 cell cultures transduced with lentiviral constructs expressing aPKC mutants and already selected in blasticidin, were grown on Transwell inserts (6.5 mm diameter). After two days of culture, the cells were transduced with an inducible NF-κB-responsive firefly luciferase reporter (Qiagen, CLS-013L, Valencia CA) for 24 hours and puromycin-selected for 8 days. Blasticidin selection was maintained during this period as well. For detection, cells were extracted in PBS and loaded into a 96-well microplate. The extracts were combined (1:1) with Bright-Glo Luciferase Assay Substrate (Promega, E2610, Madison WI) and incubated for two minutes at room temperature. Luminescence detection was performed with a Synergy H1 Multi-Mode Microplate Reader (BioTek, Winooski, VT). Data were collected using Gen5.2.00 software BioTek and normalized by protein content. Band densitometry in chemiluminiscent immunoblots was performed with a VersaDoc 5000MP gel imager (BioRad) using Quantity One software.

### Quantitative real-time polymerase chain reaction (qPCR)

RNA was isolated using the RNeasy Plus Mini Kit (QIAGEN, Hilden, Germany) and reverse-transcribed using the iScript cDNA Synthesis Kit (BIO-RAD, Hercules, CA, USA). IL-8 and GRO-1 -3 gene expression was quantified by Quantitative PCR analysis using TaqMan Gene Expression Assays (Applied Biosystems. Foster City, CA, USA) on an iCycler instrument (Bio-Rad, Hercules, CA, USA). RNA expression levels were normalized to internal control GAPDH. To compare the transcript levels between different samples the ΔΔCT comparative cycle threshold calculation was used (Livak and Schmittgen, 2001).

### Immunofluorescence and image analysis

Immunofluorescence images were acquired with a Leica SP5 confocal microscope, under 63× oil immersion (NA 1.4) objective, using Leica LAS software. Images are shown as single confocal sections, except for DAPI nuclear signal which was often at a different focal plane and is presented as stack maximum projections. Pixel intensity measurements were performed as described before (Wald et al., 2011).

### Statistics

Average and standard deviation were used. Differences between averages were analyzed by Student's t test. For nuclear/cytoplasm signal ratios, descriptive statistics were used showing data in the form of box-and-whisker plots (McGill et al., 1978). In this case, significance of the difference between groups was analyzed by Kruskal-Wallis test (Kruskal and Wallis, 1952).
