# Par-complex aPKC and Par3 cross-talk with innate immunity NF-κB pathway in epithelial cells

**DOI:** 10.1242/bio.20137260

**Published:** 2013-12-05

**Authors:** Radia Forteza, Flavia A. Wald, Anastasia Mashukova, Zhanna Kozhekbaeva, Pedro J. Salas

This Corrigendum relates to *Biol. Open* 2013, 2:1264–1269.

Unfortunately one of the author affiliations in this article was incorrect. The correct address for Anastasia Mashukova is as follows: Department of Physiology, College of Medical Sciences, Nova Southeastern University, Fort Lauderdale-Davie, FL 33328, USA.

